# The prevalence of HPV among 164,137 women in China exhibited some unique epidemiological characteristics

**DOI:** 10.1186/s13027-023-00553-4

**Published:** 2023-11-10

**Authors:** Qiong Chen, Wanglei Qu, Yu Zhao, Li Shu, Yi Wang, Xiangnan Chen

**Affiliations:** 1 Department of Obstetrics and Gynecology, The Second Affiliated Hospital and Yuying Children’s Hospital of Wenzhou Medical University, Wenzhou, Zhejiang China; 2 Department of Laboratory Medicine, The Second Affiliated Hospital and Yuying Children’s Hospital of Wenzhou Medical University, No. 1111 East Section of Wenzhou Avenue, Longwan District, Wenzhou, Zhejiang China

**Keywords:** Human papillomavirus, Cervical cancer, High-risk HPV

## Abstract

**Objective:**

The persistence of HPV infection is a significant etiological factor in the development of cervical cancer. The present study investigated the prevalence and genotype distribution of human papillomavirus (HPV) in a cohort of 164,137 unvaccinated women from Wenzhou, aiming to provide guidance for clinics in the cervical cancer screening and HPV vaccination strategies.

**Methods:**

The present retrospective study included a total of 164,137 women, comprising 118,484 outpatients and 45,653 healthy female subjects recruited from 2015 to 2020. Cervical exfoliated cells were collected from these participants for subsequent DNA extraction. The extracted DNA samples were underwent analysis using a fluorescence in situ hybridization method, encompassing the detection of 27 HPV genotypes.

**Results:**

The overall prevalence of HPV was 17.35%; this corresponded to a prevalence of 19.10% in the outpatient group and 12.82% in the healthy female group. Among the outpatient group, the five most prevalent HPV genotypes were identified as HPV 52, 58, 16, 53, and 61. In the healthy female group, the five most common HPV genotypes were found to be HPV 52, 53, 58, 61, and 81. Additionally, it was estimated that the highest rate of HPV infection occurred among individuals aged between 10 and 19 years old (44.65%) and those aged between 60 and 69 years old (27.35%).

**Conclusions:**

The prevalence of HPV in this region is substantial; therefore, it is imperative to implement scientifically sound and rational clinical interventions such as vaccination. Routine cervical screening should be performed to prevent the development of cervical intraepithelial neoplasia resulting from persistent infection with high-risk HPV, particularly in women with gynecological diseases and those over 60 years old.

**Supplementary Information:**

The online version contains supplementary material available at 10.1186/s13027-023-00553-4.

## Background

Human papillomavirus (HPV) is one of the most common sexually transmitted infections that primarily targets the human epithelium [1]. More than 200 genotypes of HPV have been identified and categorized as low-risk HPV (LR-HPV) or high-risk HPV (HR-HPV) [2]. Infections with the LR-HPV types, such as HPV 6 and 11, result in the formation of predominantly clinically apparent benign lesions known as genital warts. Persistent infection with HR-HPV leads to the progression towards cervical cancer, which is the fourth most common cancer among women globally [3]. Based on epidemiological data, the International Agency for Research on Cancer (IARC) categorized HPV 16, 18, 26, 31, 33, 35, 39, 45, 51, 52, 53, 56, 58, 59, 66, 68, and 82 as carcinogenic or probably carcinogenic [4]. It is known that HPV16 and 18 have the highest risk of causing cervical cancer, followed by HPV52 [5].The total direct medical expenditures associated with cervical cancer prevention and treatment have been estimated at approximately $6 billion in the United States of America [6]. According to the latest global burden study on cancer, there were an estimated 110,000 new cases of cervical cancer and 59,000 deaths in China in 2020 [3].

The incidence of cervical cancer is significantly elevated in countries burdened with a high prevalence of HPV. Consequently, the implementation of HPV detection and vaccination programs has been widely recognized as the most efficacious approach to prevent cervical cancer [7]. However, only a mere 3% of Chinese women aged 9–45 had received a comprehensive HPV vaccinationin 2020 [8]. Data on the prevalence and genotype distribution of HPV can serve as a fundamental basis for estimating HPV infection and developing scientifically sound and effective strategies for HPV vaccination.

In China, cervical cancer screening procedures are based on guidelines published by Colposcopy and Cervical Pathology (ASCCP) and Society of Gynecologic Oncology (SGO), which include co-testing with cytology and HPV detection [9]. Recent studies in China have revealed significant regional and demographic variations in the prevalence of HPV, ranging from 15.5% to 28.95%; the three most prevalent genotypes were identified as HPV52, 16 and 58 [10–16]. However, the majority of these studies have primarily focused on single healthy females or individuals medical care at hospitals. This study represents a pioneering effort in comparing HPV prevalence and genotype distribution among carriers of gynecological diseases and healthy females, thereby providing invaluable data for comprehensive systematic and epidemiological assessments of HPV, as well as the development of effective clinical screening protocols for cervical cancer.

## Methods

### Study population

From January 2015 to December 2020, we collected a total of 164,137 participants (age range 12–89 years) refraining from receiving HPV vaccination in this retrospective study. The participants were categorized into two distinct groups: (1) The outpatients group (OG) including 118,484 outpatients from the department of gynecology underwent HPV tests for various indications, including vaginitis, urethritis, irregular vaginal bleeding, cervicitis, undiagnosed abdominal pain, and genital warts, and women with known HPV infection were excluded from the group. (2) The healthy female group (HFG) consisted of 45,653 female subjects without reported gynecological symptoms who visited the physical examination center. The participants were recruited from the Second Affiliated Hospital and Yuying Children’s Hospital of Wenzhou Medical University.

### Sample collection and HPV genotyping

Cervical exfoliated cells were obtained from women using a cytobrush (Shanghai Tellgen Life Technologies Inc.) for genomic DNA extraction. DNA was extracted and purified using a commercial kit (Shanghai Tellgen Life Technologies Inc.) by the exchange resin adsorption method. The method was performed according to the manufacturer’s instructions. Subsequently, HPV DNA was detected and genotyped by flow-through hybridization and gene chip of Tellgenplex^®^HPV 27 genotyping Assay (CFDA20173404697, Shanghai Tellgen Life Technologies Inc.) using Luminex 200 System (Luminex Corporation in the USA), following the manufacturer’s instructions. This method uses multiplex PCR amplification with universal primers and flow fluorescence hybridization to simultaneously detect 27 types of HPV, including 10 LR-HPV genotypes (6, 11, 40, 42, 43, 44, 55, 61, 81, and 83), and 17 HR-HPV genotypes (16, 18, 26, 31, 33, 35, 39, 45, 51, 52, 53, 56, 58, 59, 66, 68, and 82). The advantages of the flow fluorescence hybridization method used in the present study have been confirmed in previous studies [17].

Positive and negative controls were used during testing to verify the reliability of the results. The β-globin gene was utilized as the internal reference sequence, and a signal value < 1000 was considered an unqualified detection. According to the manufacturer’s verification report, when compared to the Sanger sequencing method as the reference method, this assay would have a sensitivity of 100%, specificity of 76.33%, and total coincidence rate of 96.08%.

The positive specimens included in the study underwent HPV typing, and overall prevalence of HPV was calculated. Additionally, an analysis of the annual trend in overall prevalence was conducted, along with calculations for prevalence of both multiple and single infection, HR-HPV and LR-HPV infection, as well as proportions of infection within each age group. Furthermore, a comparison between the OG group and HFG group was performed to identify any differences. The genotyping of all HPV infections was statistically analyzed, and the above indicators were also analyzed.

Statistical analysis was performed using WPS Office (Kingsoft Office, Inc.) and GraphPad Prism 5 (GraphPad Software, Inc.). The differences in the HPV positive rates and proportions of multiple-type HPV infections between the OG and HFG were assessed by the unpaired Student’s t-test. A statistically significant difference was considered when *P* < 0.01.

## Results

### Overall and type-specific HPV prevalence

A total of 164,137 women were involved in the present study, including 118,484 females in the OG and 45,653 females in the HFG (Table 1). The overall prevalence of HPV was 17.35%, with 28,480 out of the total 164,137 cases being positive for HPV among women. The positive cases accounted for 19.10% (22,626/118,484) of the OG and 12.82% (5854/45,653) of the HFG, respectively. The prevalence of HPV in the OG was significantly higher than that of the HFG (*P* < 0.01).

**Table 1:** HPV genotype distribution in the total population, the OG, and the HPG

Genotype	Total (n = 164,137)		OG(n = 118,484)		HPG (n = 45,653)		*P*
	n	P%	n	P%	n	P%	
HR-HPV	26,568	16.19	21,424	18.08	5144	11.27	< 0.001
HPV 16	2746	1.67	2371	2.00	375	0.82	< 0.001
HPV 18	1473	0.90	1215	1.03	258	0.57	< 0.001
HPV 26	41	0.02	36	0.03	5	0.01	> 0.001
HPV 31	467	0.28	394	0.33	73	0.16	< 0.001
HPV 33	853	0.52	693	0.58	160	0.35	< 0.001
HPV 35	612	0.37	483	0.41	129	0.28	< 0.001
HPV 39	1947	1.19	1546	1.30	401	0.88	< 0.001
HPV 45	372	0.23	301	0.25	71	0.16	< 0.001
HPV 51	1442	0.88	1158	0.98	284	0.62	< 0.001
HPV 52	5397	3.29	4259	3.59	1138	2.49	< 0.001
HPV 53	3070	1.87	2363	1.99	707	1.55	< 0.001
HPV 56	1425	0.87	1134	0.96	291	0.64	< 0.001
HPV 58	3220	1.96	2663	2.25	557	1.22	< 0.001
HPV 59	1227	0.75	986	0.83	241	0.53	< 0.001
HPV 66	1211	0.74	997	0.84	214	0.47	< 0.001
HPV 68	629	0.38	469	0.40	160	0.35	> 0.001
HPV 82	436	0.27	356	0.30	80	0.18	< 0.001
LR-HPV	10,538	6.42	8279	6.99	2259	4.95	< 0.001
HPV 06	862	0.53	725	0.61	137	0.30	< 0.001
HPV 11	484	0.29	436	0.37	48	0.11	< 0.001
HPV 40	169	0.10	132	0.11	37	0.08	> 0.001
HPV 42	603	0.37	465	0.39	138	0.30	> 0.001
HPV 43	1302	0.79	1044	0.88	258	0.57	< 0.001
HPV 44	1379	0.84	1067	0.90	312	0.68	< 0.001
HPV 55	1170	0.71	891	0.75	279	0.61	> 0.001
HPV 61	2204	1.34	1686	1.42	518	1.13	< 0.001
HPV 81	2137	1.30	1648	1.39	489	1.07	< 0.001
HPV 83	228	0.14	185	0.16	43	0.09	> 0.001

*n* Number of cases, *P*% Prevalence rate, *HR* High risk, *LR* Low risk, *HPV* Human papillomavirus, *OG* Outpatient group, *HPG* Healthy female group

A total of 27 HPV genotypes were identified in the present study. The total number of positive HPV genotypes was 37,106, among which 29,703 were from the OG, with a per capita carrying rate of 0.25 (29,703/118,484). A total of 7403 positive HPV genotypes were noted in the HFG, with a per capita carrying rate of 0.16 (7403/45,653).

**Fig. 1:**
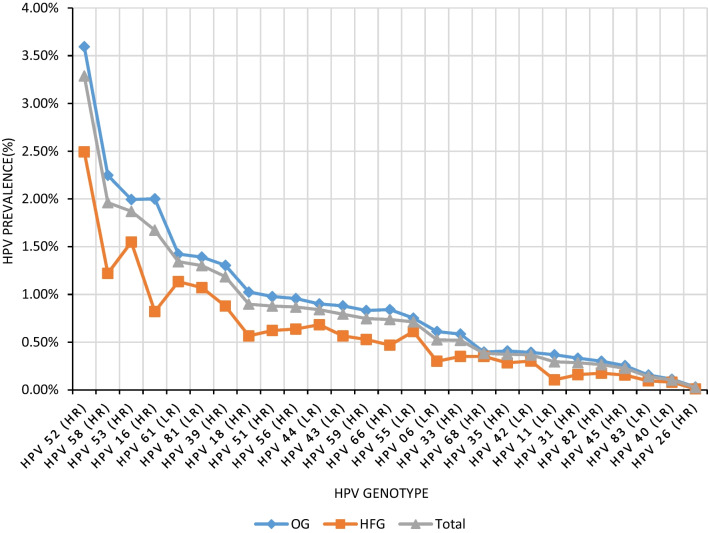
The prevalence of HPV genotypes ranked from highest to lowest. The abscissa was sorted by HPV prevalence in total from high to low. *HPV* Human papillomavirus

**Fig. 2:**
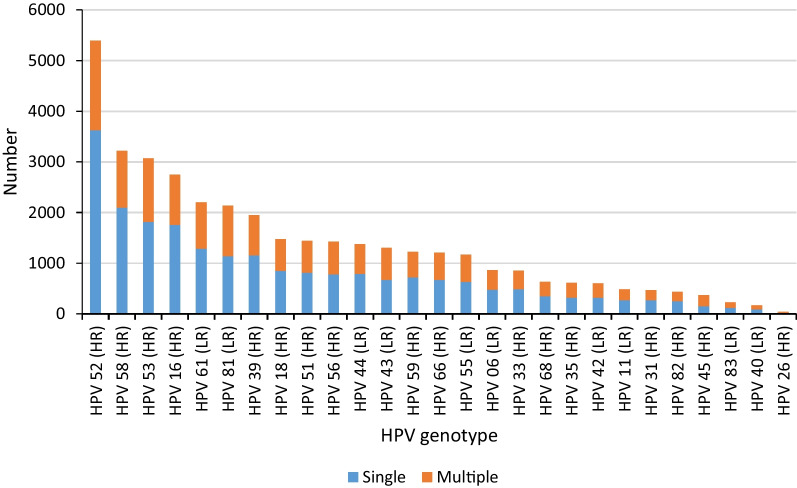
Comparison of the number of multiple infections and single infections

In total, HPV 52 (3.29%), 58 (1.96%), 53 (1.87%), 16 (1.67%), and 61 (1.34%) were the five most prevalent HPV genotypes. The five most common genotypes were HPV 52, 58, 16, 53, and 61 in the OG, and the five most common genotypes were HPV 52, 53, 58, 61, and 81 in the HFG (Fig. 1). The percentage of positive detection for each HPV type was analyzed over a total period of 5 years (Fig. 2). The six most common genotypes, namely HPV 52 (14.54%), 58 (8.68%), 53 (8.27%), 16 (7.40%), 61 (5.94%), and 81 (5.76%), accounted for approximately 50% of all infection types (Additional file 1: Fig. S1).

### Genotype-specific prevalence of HR-HPV

HR-HPV was detected in 26,568 individuals, with a total infection rate of 16.19% (26,568/164,137) (Table 1). The HR-HPV infection rate of the OG and the HFG was 18.08% (21,424/118,484) and 11.27% (5144/45,653), respectively. The HR-HPV infection rate of the OG was significantly higher than that of the HFG (*P* < 0.01).

The most prevalent HR-HPV genotypes were HPV 52 (3.29%, 5397/164,137), followed by HPV 58 (1.96%, 3220/164,137), HPV 53 (1.87%, 3070/164,137), HPV 16 (1.67%, 2746/164,137), and HPV 39 (1.19%, 1947/164,137).

### Distribution characteristics of single and multiple HPV infections

The frequency and number of HPV infections are shown in Table 2. Among women with a single HPV infection, HR-HPV infection accounted for 74.33% (12,867/17,286) in the OG and for 70.33% (3273/4,654) in the HFG (*P* > 0.01). Among women with multiple HPV infections, the multiple infections with the HR-HPV rate accounted for 93.39% (4987/5340) of the population in the OG and for 92% (1104/1200) of the population in the HFG (*P* > 0.01).

**Table 2:** Frequency and prevalence of single and multiple HPV infection. HPV, human papillomavirus

Variables	Total (n = 164,137)		OG (n = 118,484)		HPG (n = 45,653)		*P* value
	n	P%	n	P%	n	P%	
Single	21,940	13.37	17,286	14.59	4654	10.19	< 0.001
Double	4989	3.04	4056	3.42	933	2.04	< 0.001
Triple	1156	0.70	954	0.81	202	0.44	< 0.001
Quadruple	294	0.18	244	0.21	50	0.11	< 0.001
Quintet	72	0.04	59	0.05	13	0.03	> 0.001
Sextuple	29	0.02	27	0.02	2	0.00	> 0.001

*n* Number of cases, *P*% Prevalence rate

Multiple and single HPV genotypes accounted for 40.87% (15,166/37,106) and 59.13% (21,940/37,106) of all positive types, respectively. Single infections and multiple infections were identified across all genotypes (Fig. 2). The prevalence of HPV 16, 39, 52, and 58 in the single infection group was higher than that of the multiple infection group (*P* < 0.01).

### Time trends of prevalence and genotype of HPV

**Fig. 3:**
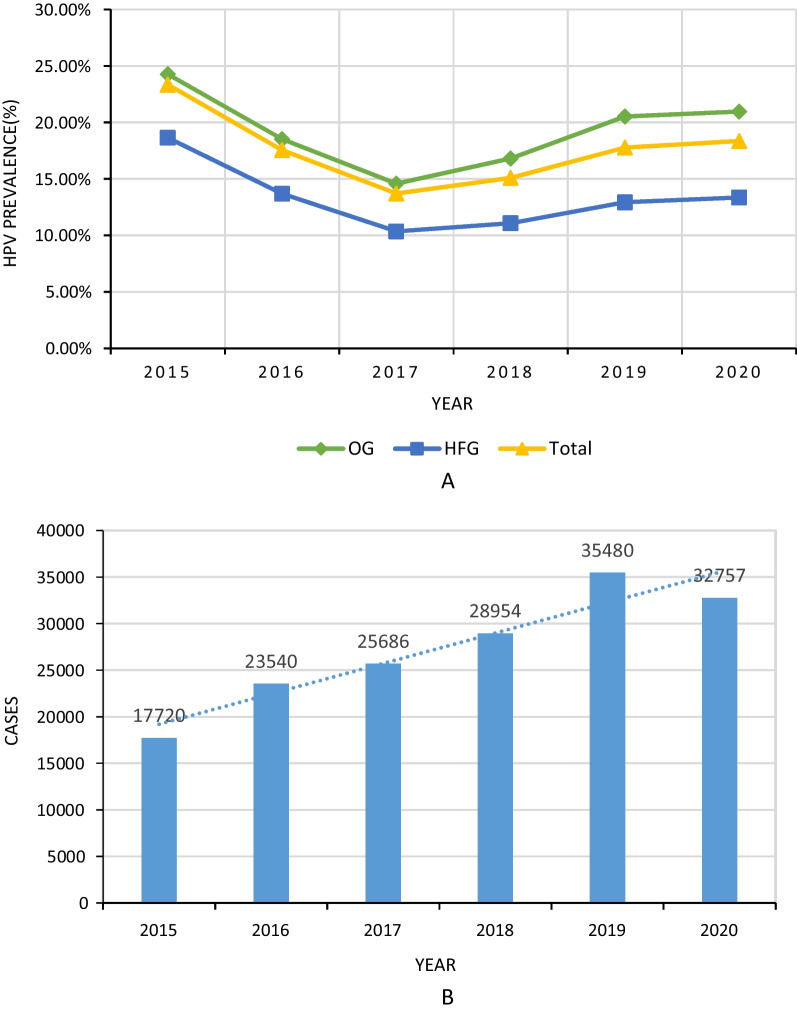
Summary of HPV testing volume and infection rate from 2015 to 2020. **A** Trend of HPV infection in each group. **B** The number of HPV genotyping tests during the period 2015–2020. *HPV* Human papillomavirus

The prevalence of HPV in the OG and HFG indicated almost the same trend (Fig. 3A). The total prevalence of HPV indicated a significant decrease from 23.35% in 2015 to 13.71% in 2017. Subsequently, the prevalence slowly increased to 18.35% in 2020. The number of HPV genotyping tests increased every year during the period 2015–2019. However, the COVID-19 lockdown in 2020 resulted in a reduction in the quantity of HPV testing (Fig. 3B).

**Fig. 4:**
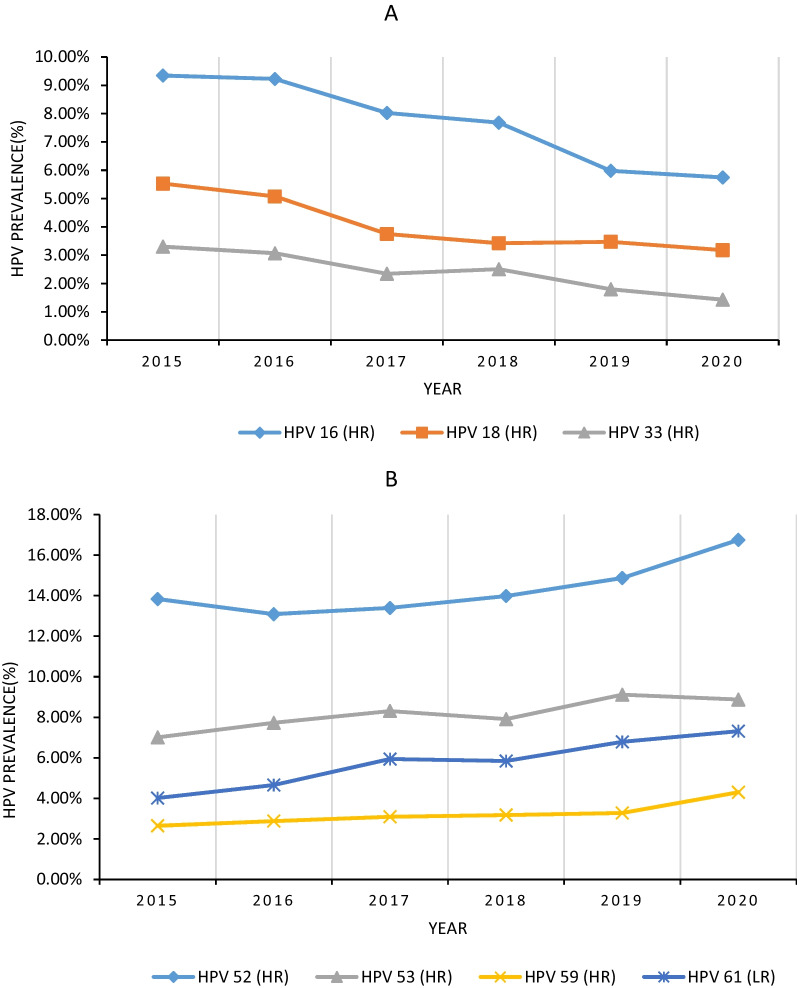
Time trends of specific genotypes of HPV. **A** HPV 16, 18, and 33 indicated a downward trend. **B** HPV 52, 53, 59 and 61 indicated an upward trend. HPV, human papillomavirus

The majority of viral infection types were consistent with the overall trend of HPV-positive infections; however, certain genotypes demonstrated an annual fluctuation in prevalence (Fig. 4).

### Age-specific prevalence and genotype of HPV

**Fig. 5:**
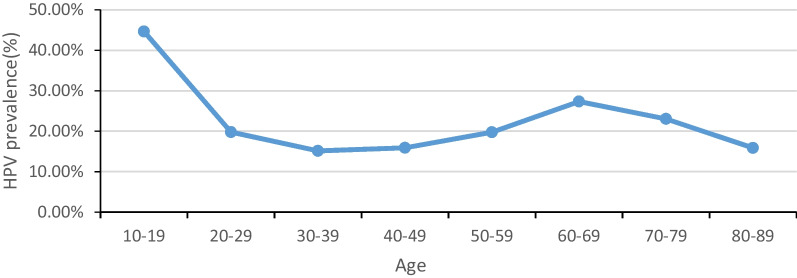
The overall age-specific prevalence of HPV. HPV, human papillomavirus

All the participants were stratified into 8 groups based on their age (Table 3). The overall age-specific prevalence of HPV exhibited bimodal distribution, with two distinct peaks observed. The infection rates for the specific age groups were as follows: 44.65%, 19.79%, 15.14%, 15.90%, 19.76%, 27.35%, 23.04%, and 15.87% (Fig. 5). The first peak corresponded to 44.65% in the 10–19 age group. The prevalence of HPV decreased to 19.79% in the 20–29 age group and continuously increased to 27.35% in the 60–69 age group. The lowest prevalence (below 16%) was observed in the age group of 30–49, with a predominant representation of participants within this specific age range. Two infection peaks corresponding to subjects under 20 and between 60 and 69 years of age were noted in the OG, whereas only one infection peak was identified in the HFG among individuals aged 60–79.

**Table 3:** Summary of HPV infection in the OG and HPG at each age group

Age group	Cases	Positive cases	Positive rate	Multiple infection cases	Multiple infection rate	HR-HPV infection cases	HR-HPV infection rate	Percentage of HR-HPV in positive cases
*OG*								
10–19	301	140	46.51	61	20.27	113	37.54	80.71
20–29	16,940	3559	21.01	1035	6.11	2888	17.05	81.15
30–39	40,785	6868	16.84	1445	3.54	5580	13.68	81.25
40–49	38,732	6747	17.42	1328	3.43	5147	13.29	76.29
50–59	16,452	3655	22.22	950	5.77	2800	17.02	76.61
60–69	4403	1440	32.70	455	10.33	1148	26.07	79.72
70–79	758	197	25.99	62	8.18	159	20.98	80.71
80–89	113	20	17.70	4	3.54	19	16.81	95.00
Total	118,484	22,626	19.10	5340	4.51	14,358	12.12	63.46
*HPG*								
10–19	17	2	11.76	1	5.88	1	5.88	50.00
20–29	3355	457	13.62	127	3.79	370	11.03	80.96
30–39	14,945	1572	10.52	278	1.86	1196	8.00	76.08
40–49	14,949	1788	11.96	285	1.91	1297	8.68	72.54
50–59	8634	1301	15.07	282	3.27	927	10.74	71.25
60–69	3133	621	19.82	195	6.22	496	15.83	79.87
70–79	544	103	18.93	31	5.70	82	15.07	79.61
80–89	76	10	13.16	1	1.32	8	10.53	80.00
Total	45,653	5854	12.82	1200	2.63	3274	7.17	55.93

*HPV* Human papillomavirus, *OG* Outpatient group, *HPG* Healthy female group

The age-specific percentage of the HPV genotype in the positive cases was analyzed (Additional file 1: Table S1). The data indicated that young subjects under the age of 20 infected with HPV 6, 11, 16, 18, and 52 were the main subjects infected, accounting for approximately half of the total infection cases. The infection rates of HPV 6 and 11 were significantly higher in the aforementioned age group (< 20 years of age) than those of the other age groups, while HPV 53 infected a significantly lower number of subjects in the < 20-year group compared with that of the other age groups.

**Fig. 6:**
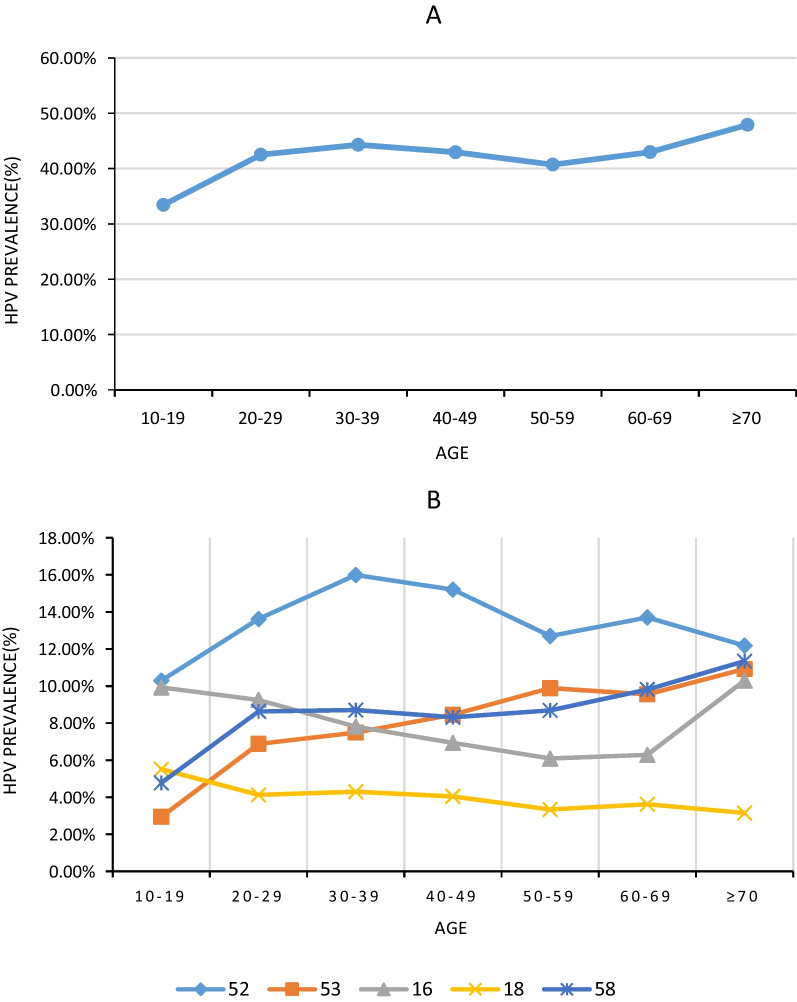
Age-specific prevalence of the six most common HR-HPV genotypes. **A** The overall prevalence of HPV 52, 58, 53, 16, 39, and 18 was gradually increased with age. **B** HPV 52 indicated an apparent bimodal pattern, while HPV 18 hardly changed with age. HPV 53 and 58 increased with age, while HPV 16 decreased with age until 70. *HR-HPV* High-risk HPV, *HPV* Human papillomavirus

The overall risk of the six most common HR-HPV genotypes (HPV 52, 58, 53, 16, 39, and 18) exhibited an age-dependent increase. However, individual genotypes displayed distinct age-related trends (Fig. 6).

## Discussion

The incidence and mortality rates of cervical cancer in China rank second globally, while the HPV vaccination coverage remains as low as 3%, highlighting an urgent need for enhanced immunization coverage. HPV prevalence data are crucial in guiding effective HPV vaccination strategies and facilitating early prevention of cervical cancer. The current retrospective study presents the prevalence and distribution of HPV among 164,137 women, including outpatients and healthy females, between 2015 and 2020 in Wenzhou, a coastal city of 9 million people located in southeast China. Our data revealed a persistent high level of HPV infection, with an overall prevalence of HPV was 17.35%. Notably, the prevalence of HPV in the OG was approximately 6% higher than that in the HFG. This data supports the notion that gynecological diseases can disrupt the vaginal microecological environment and increase to susceptibility to HPV [18].

According to previous reports, HPV prevalence ranges from 6.70 to 44.50% in China, reflecting the vast geographical expanse and varying levels of economic development within the country [19, 20]. The present study adopted the same detection method in the same area for five years, providing solid evidence of HPV epidemiology in the region. And we revealed that the trend of HPV infection decreased from 23.35% in 2015 to 13.71% in 2017; subsequently, it increased to 18.35% in 2020. We posit that the level of socioeconomic development constitutes the primary determinant influencing variations in HPV infection trends during this time period. Correspondingly, economically advanced regions in China, such as Shanghai, Guangdong, and Shenzhen, exhibit a generally low HPV infection, specifically recording rates of 15.5%, 19.9%, and 18.7% respectively [21–23]. However, prior to 2016, most cities exhibited higher rates exceeding 20% or even exceeding 30%. For instance, Harbin, Henan, Fujian, Jiangxi, Zhejiang, and Chongqing report respective infection rates of 36.45%, 23.98%, 22.5%, 22.49%, 20.54%, and 26.2% [24–28]. Nevertheless, economically developed areas still exhibit persistently high HPV infection rates above 15% in China, underscoring the urgent necessity for comprehensive vaccination strategies aimed at mitigating these prevalence levels.

Worldwide meta-analysis studies indicate that the five most common infection types are HPV16, 18, 52, 31, and 58 [29]. However, the current study identified HPV 52, 58, 53, 16, and 39 as the five most common HR-HPV genotypes. We have observed an increased trend in HPV 53 and 59 infections, which are not covered by the nine-valent HPV vaccine. The IARC designates HPV 53 as group 2B (possibly carcinogenic), indicating a potential association with a low incidence of cervical cancer [4]. However, given the high prevalence of HPV 53 in China, it is imperative to allocate significant attention to this viral strain and consider its inclusion in the development of next-generation vaccines.

In 2015, interim guidelines were issued by Colposcopy and Cervical Pathology (ASCCP) and the Society of Gynecologic Oncology (SGO) recommending the discontinuation of cervical screening after the age of 65. Several studies have also demonstrated a significant decline in HR-HPV infection rates among individuals aged above 45 [30, 31]. However, contrary to these findings, our study identified an increased prevalence of HPV among women aged above 40, with those between ages 60–69 exhibiting the highest rates. Factors such as continued active sexual activity and weakened immune system may contribute to the elevated risk of HPV infection in elderly women. Furthermore, there is evidence suggesting that HPV increases the likelihood of developing cervical neoplasia in older females (aged around or above forty) [32]. Based on our comprehensive data analysis, we recommend cervical screenings for all females aged above forty and particularly emphasize regular testing for those aged sixty or older.

In this study, a total of 164 137 women underwent HPV testing and were stratified accordingly. The prevalence of HPV was compared between carriers of gynecological diseases and healthy individuals, while also analyzing the infection rates across different types and age groups. This comprehensive dataset on HPV infection among women in our region provides valuable insights for HPV screening and vaccination strategies. However, we did not conduct a detailed categorization of patients based on specific clinical disease types to enable a comparison of HPV infection rates within these categories, which would have facilitated the exploration of the association between HPV infection and gynecological diseases. The present study revealed a declining trend of HPV 16 prevalence with increasing age, while HPV 52 demonstrated a bimodal distribution pattern characterized by peaks at ages 30–39 and 60–69 years. In future investigations, data needs to be gathered in order to explore and elucidate the association between HPV infection and gynecological diseases, as well as the interplay among HPV genotypes, age demographics, and immune responses.

## Conclusions

The present study revealed the temporal trends and age-specific genotype distribution of HPV in Wenzhou. Despite advancements in social and economic development, the prevalence of HPV remains high. We strongly advocate for the prompt and comprehensive implementation of targeted measures, such as vaccination. Notably, the OG group exhibited a significantly higher prevalence of HPV compared to the HFG, underscoring the necessity for routine cervical cancer screening among patients with gynecological diseases. Furthermore, there is an escalating prevalence of HR-HPV infections among women aged over 40 years old that peaks after reaching 60 years old. Consequently, this study strongly advocates for regular cervical screening in women above the age of 60 to mitigate the risk of developing cervical intraepithelial neoplasia subsequent to persistent HPV infection.

### Supplementary Information

**Additional file 1: Figure S1.** The proportion of each HPV type corresponding to a positive detection during a period of 5 years. *HPV* Human papillomavirus. **Table S1.** Age-specific genotypes of HPV infection. *HPV* Human papillomavirus.

## Data Availability

The data generated in the present study may be requested from the corresponding author.

## Abbreviations

HPV: Human papillomavirus
LR-HPV: Low-risk HPV
HR-HPV: High-risk HPV
OG: Outpatient group
HFG: Healthy female group

## References

[1] Dunne EF, Park IU (2013). HPV and HPV-associated diseases. Infect Dis Clin N Am.

[2] Olusola P, Banerjee HN, Philley JV, Dasgupta S (2019). Human papilloma virus-associated cervical cancer and health disparities. Cells..

[3] Sung H, Ferlay J, Siegel RL, Laversanne M, Soerjomataram I, Jemal A (2021). Global cancer statistics 2020: GLOBOCAN estimates of incidence and mortality worldwide for 36 cancers in 185 countries. CA Cancer J Clin.

[4] Wang R, Pan W, Jin L (2020). Human papillomavirus vaccine against cervical cancer: opportunity and challenge. Cancer Lett.

[5] Du H, Luo H, Wang C, Qu X, Belinson JL, Wu R (2021). The prevalence of HR-HPV infection based on self-sampling among women in China exhibited some unique epidemiologic features. J Clin Epidemiol.

[6] Armstrong EP (2010). Prophylaxis of cervical cancer and related cervical disease: a review of the cost-effectiveness of vaccination against oncogenic HPV types. J Manag Care Pharm.

[7] Luo LP, He P, Liu QT (2021). Prevalence and genotype distribution of HPV infection among 214,715 women from Southern China, 2012–2018: baseline measures prior to mass HPV vaccination. BMC Infect Dis..

[8] Wang L, Zhong Y, Di J (2021). Current experience in HPV vaccination in China. Indian J Gynecol Oncol.

[9] Bhatla N, Singhal S (2020). Primary HPV screening for cervical cancer. Best Pract Res Clin Obstet Gynaecol.

[10] Huang W, Xu H, Hu H (2022). The prevalence of human papillomavirus among women in northern Guangdong Province of China. Sci Rep..

[11] Zhang H, Zhang S (2023). Prevalence and genotype distribution of human papillomavirus infection among female outpatients in Northeast China: a population-based survey of 110,927 women. Arch Gynecol Obstet.

[12] Zhang Y, Xu Y, Dian Z (2022). Prevalence and genotype distribution of human papillomavirus infection among 40,613 women: an outpatient-based population study in Kunming, Yunnan. Front Public Health.

[13] Wei X, Lu Q, Wang S (2022). Prevalence characteristics of cervical human papillomavirus genotypes in Nanning, China: a 10-year survey of 77,756 women from one medical center. J Med Virol.

[14] Wei L, Ma L, Qin L, Huang Z (2022). The prevalence and genotype distribution of human papillomavirus among women in Guangxi, southern China. Infect Agent Cancer.

[15] Zhu Y, Qian F, Zou W (2021). Prevalence and genotype distribution of human papillomavirus infection in Huzhou City, eastern China, 2018–2019. Trans R Soc Trop Med Hyg.

[16] Yu H, Yi J, Dou YL, Chen Y, Kong LJ, Wu J (2021). Prevalence and genotype distribution of human papillomavirus among healthy females in Beijing, China, 2016–2019. Infect Drug Resist.

[17] He L, He J (2019). Distribution of high-risk HPV types among women in Sichuan province, China: a cross-sectional study. BMC Infect Dis..

[18] Liang Y, Chen M, Qin L, Wan B, Wang H (2019). A meta-analysis of the relationship between vaginal microecology, human papillomavirus infection and cervical intraepithelial neoplasia [published correction appears in Infect Agent Cancer. 2019 Dec 9;14:47]. Infect Agent Cancer.

[19] Li K, Li Q, Song L, Wang D, Yin R (2019). The distribution and prevalence of human papillomavirus in women in mainland China. Cancer.

[20] Zhang J, Cheng K, Wang Z (2020). Prevalence and distribution of human papillomavirus genotypes in cervical intraepithelial neoplasia in China: a meta-analysis. Arch Gynecol Obstet.

[21] Li H, Li P, Huang L, Sun L, Ren H, Li P (2020). Prevalence characteristics of cervical human papillomavirus (HPV) infection in the Zhoupu District, Shanghai City, China. Virol J.

[22] Zhao P, Liu S, Zhong Z (2018). Prevalence and genotype distribution of human papillomavirus infection among women in northeastern Guangdong Province of China. BMC Infect Dis..

[23] Chen T, Cai S, Lin J (2022). Prevalence and genotype distribution of human papillomavirus among 29 263 women from the Longgang community of Shenzhen. Trans R Soc Trop Med Hyg.

[24] Sun B, He J, Chen X (2014). Prevalence and genotype distribution of human papillomavirus infection in Harbin, Northeast China. Arch Virol.

[25] Wang XC, Sun LQ, Ma L (2014). Prevalence and genotype distribution of human papillomavirus among women from Henan. China Asian Pac J Cancer Prev.

[26] Wu C, Zhu X, Kang Y (2017). Epidemiology of Humanpapilloma virus infection among women in Fujian, China [published correction appears in BMC Public Health. 2017 Sep 22;17 (1):736]. BMC Public Health..

[27] Zhong TY, Zhou JC, Hu R (2017). Prevalence of human papillomavirus infection among 71,435 women in Jiangxi Province. China J Infect Public Health.

[28] Chen X, Xu H, Xu W (2017). Prevalence and genotype distribution of human papillomavirus in 961,029 screening tests in southeastern China (Zhejiang Province) between 2011 and 2015. Sci Rep.

[29] Bruni L, Diaz M, Castellsagué X, Ferrer E, Bosch FX, de Sanjosé S (2010). Cervical human papillomavirus prevalence in 5 continents: meta-analysis of 1 million women with normal cytological findings. J Infect Dis.

[30] Babi A, Issa T, Issanov A (2021). Prevalence of high-risk human papillomavirus infection among Kazakhstani women attending gynecological outpatient clinics. Int J Infect Dis.

[31] Aimagambetova G, Babi A, Issanov A (2021). The distribution and prevalence of high-risk HPV genotypes other than HPV-16 and HPV-18 among women attending gynecologists’ offices in Kazakhstan. Biology (Basel).

[32] Gilham C, Crosbie EJ, Peto J (2021). Cervical cancer screening in older women. BMJ.

