# Supplementary material for: The prevalence of HPV among 164,137 women in China exhibited some unique epidemiological characteristics
Source: Infect Agent Cancer. 2023 Nov 10;18:72. doi: 10.1186/s13027-023-00553-4 (PMC10638728; doi:10.1186/s13027-023-00553-4)
Supplement: Supplementary file 1 — Additional file 1: Figure S1. The proportion of each HPV type corresponding to a positive detection during a period of 5 years. HPV Human papillomavirus. Table S1. Age-specific genotypes of HPV infection. HPV Human papillomavirus. [file 13027_2023_553_MOESM1_ESM.docx]

Supplementary Figure 1. The proportion of each HPV type corresponding to a positive detection during a period of 5 years. HPV, human papillomavirus.


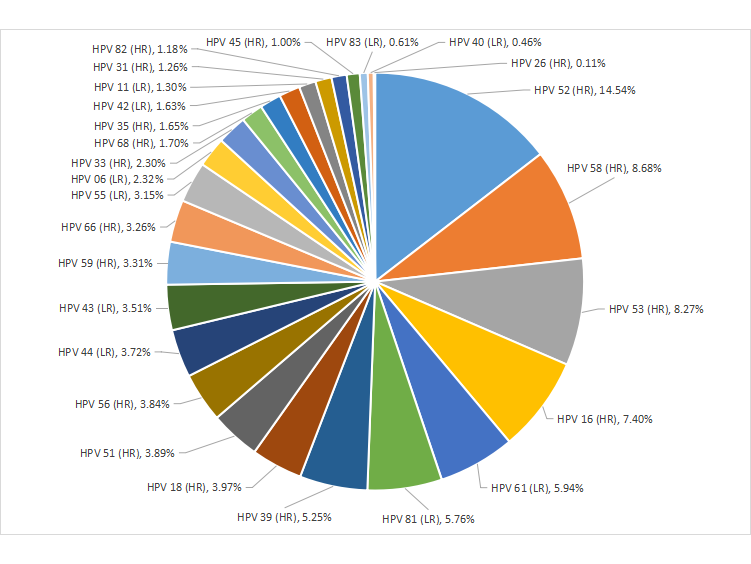


Supplementary Table 1. Age-specific genotypes of HPV infection. HPV, human papillomavirus.

| Genotype | 10-19 | | 20-29 | | 30-39 | | 40-49 | | 50-59 | | 60-69 | | >70 | |
| --- | --- | --- | --- | --- | --- | --- | --- | --- | --- | --- | --- | --- | --- | --- |
|  | n. | P% | n. | P% | n. | P% | n. | P% | n. | P% | n. | P% | n. | P% |
| HPV 06 | 22 | 8.09% | 230 | 4.08% | 227 | 2.14% | 212 | 2.02% | 110 | 1.67% | 48 | 1.58% | 13 | 2.73% |
| HPV 11 | 27 | 9.93% | 138 | 2.45% | 140 | 1.32% | 108 | 1.03% | 44 | 0.67% | 23 | 0.76% | 4 | 0.84% |
| HPV 16 | 27 | 9.93% | 522 | 9.25% | 829 | 7.81% | 727 | 6.93% | 401 | 6.09% | 191 | 6.29% | 49 | 10.29% |
| HPV 18 | 15 | 5.51% | 233 | 4.13% | 456 | 4.30% | 424 | 4.04% | 220 | 3.34% | 110 | 3.62% | 15 | 3.15% |
| HPV 26 | 0 | 0.00% | 6 | 0.11% | 11 | 0.10% | 14 | 0.13% | 6 | 0.09% | 4 | 0.13% | 0 | 0.00% |
| HPV 31 | 6 | 2.21% | 59 | 1.05% | 137 | 1.29% | 133 | 1.27% | 79 | 1.20% | 42 | 1.38% | 11 | 2.31% |
| HPV 33 | 7 | 2.57% | 122 | 2.16% | 221 | 2.08% | 250 | 2.38% | 139 | 2.11% | 94 | 3.10% | 20 | 4.20% |
| HPV 35 | 4 | 1.47% | 83 | 1.47% | 175 | 1.65% | 173 | 1.65% | 113 | 1.72% | 56 | 1.84% | 8 | 1.68% |
| HPV 39 | 14 | 5.15% | 330 | 5.85% | 641 | 6.04% | 508 | 4.85% | 316 | 4.80% | 131 | 4.31% | 7 | 1.47% |
| HPV 40 | 1 | 0.37% | 33 | 0.59% | 51 | 0.48% | 37 | 0.35% | 29 | 0.44% | 16 | 0.53% | 2 | 0.42% |
| HPV 42 | 2 | 0.74% | 78 | 1.38% | 116 | 1.09% | 170 | 1.62% | 170 | 2.58% | 59 | 1.94% | 8 | 1.68% |
| HPV 43 | 18 | 6.62% | 217 | 3.85% | 384 | 3.62% | 358 | 3.41% | 219 | 3.33% | 95 | 3.13% | 11 | 2.31% |
| HPV 44 | 2 | 0.74% | 123 | 2.18% | 365 | 3.44% | 504 | 4.81% | 279 | 4.24% | 97 | 3.19% | 9 | 1.89% |
| HPV 45 | 1 | 0.37% | 53 | 0.94% | 125 | 1.18% | 99 | 0.94% | 60 | 0.91% | 27 | 0.89% | 7 | 1.47% |
| HPV 51 | 12 | 4.41% | 248 | 4.40% | 480 | 4.52% | 355 | 3.39% | 224 | 3.40% | 104 | 3.43% | 19 | 3.99% |
| HPV 52 | 28 | 10.29% | 768 | 13.61% | 1697 | 15.99% | 1594 | 15.20% | 836 | 12.70% | 416 | 13.70% | 58 | 12.18% |
| HPV 53 | 8 | 2.94% | 388 | 6.88% | 795 | 7.49% | 886 | 8.45% | 651 | 9.89% | 290 | 9.55% | 52 | 10.92% |
| HPV 55 | 1 | 0.37% | 143 | 2.54% | 286 | 2.69% | 357 | 3.41% | 270 | 4.10% | 96 | 3.16% | 17 | 3.57% |
| HPV 56 | 10 | 3.68% | 209 | 3.71% | 391 | 3.68% | 389 | 3.71% | 274 | 4.16% | 139 | 4.58% | 13 | 2.73% |
| HPV 58 | 13 | 4.78% | 487 | 8.63% | 924 | 8.71% | 872 | 8.32% | 572 | 8.69% | 298 | 9.82% | 54 | 11.34% |
| HPV 59 | 18 | 6.62% | 234 | 4.15% | 338 | 3.18% | 321 | 3.06% | 221 | 3.36% | 79 | 2.60% | 16 | 3.36% |
| HPV 61 | 6 | 2.21% | 278 | 4.93% | 525 | 4.95% | 659 | 6.29% | 477 | 7.24% | 230 | 7.58% | 29 | 6.09% |
| HPV 66 | 11 | 4.04% | 218 | 3.86% | 357 | 3.36% | 304 | 2.90% | 205 | 3.11% | 97 | 3.19% | 19 | 3.99% |
| HPV 68 | 2 | 0.74% | 113 | 2.00% | 183 | 1.72% | 189 | 1.80% | 102 | 1.55% | 39 | 1.28% | 1 | 0.21% |
| HPV 81 | 8 | 2.94% | 235 | 4.17% | 563 | 5.30% | 656 | 6.26% | 444 | 6.74% | 201 | 6.62% | 30 | 6.30% |
| HPV 82 | 8 | 2.94% | 67 | 1.19% | 149 | 1.40% | 122 | 1.16% | 57 | 0.87% | 31 | 1.02% | 2 | 0.42% |
| HPV 83 | 1 | 0.37% | 26 | 0.46% | 47 | 0.44% | 63 | 0.60% | 66 | 1.00% | 23 | 0.76% | 2 | 0.42% |
| Total | 272 | 100.00% | 5,641 | 100.00% | 10,613 | 100.00% | 10,484 | 100.00% | 6,584 | 100.00% | 3,036 | 100.00% | 476 | 100.00% |
| n, number of cases; P%, percentage of positive cases in each age group. | | | | | | | | | | | | | | |
